# Prediction of Metachronous Peritoneal Metastases After Radical Surgery for Colon Cancer: A Scoring System Obtained from an International Multicenter Cohort

**DOI:** 10.1245/s10434-022-12097-9

**Published:** 2022-07-05

**Authors:** Corrado Pedrazzani, Giulia Turri, Daniele Marrelli, Hye Jin Kim, Eun Jung Park, Gaya Spolverato, Caterina Foppa, Antonino Spinelli, Salvatore Pucciarelli, Seung Hyuk Baik, Gyu Seog Choi

**Affiliations:** 1 Division of General and Hepatobiliary Surgery, Department of Surgical Sciences, Dentistry, Gynecology and Pediatrics, Verona University Hospital, University of Verona, Verona, Italy; 2 Department of Surgery, Policlinico le Scotte, University of Siena, Siena, Italy; 3 Colorectal Cancer Centre, Kyungpook National University Medical Centre, School of Medicine, Kyungpook National University, Daegu, Republic of Korea; 4 Division of Colon and Rectal Surgery, Department of Surgery, Gangnam Severance Hospital, Yonsei University College of Medicine, Seoul, Republic of Korea; 5 General Surgery 3, Department of Surgery, Oncology, and Gastroenterology, University of Padova, Padova, Italy; 6 Department of Biomedical Sciences, Humanitas University, Pieve Emanuele, Milan, Italy; 7 Division of Colon and Rectal Surgery, IRCCS Humanitas Research Hospital, Rozzano, Milan, Italy

## Abstract

**Background:**

Since novel strategies for prevention and treatment of metachronous peritoneal metastases (mPM) are under study, it appears crucial to identify their risk factors. Our aim is to establish the incidence of mPM after surgery for colon cancer (CC) and to build a statistical model to predict the risk of recurrence.

**Patients and Methods:**

Retrospective analysis of consecutive pT3–4 CC operated at five referral centers (2014–2018). Patients who developed mPM were compared with patients who were PM-free at follow-up. A scoring system was built on the basis of a logistic regression model.

**Results:**

Of the 1423 included patients, 74 (5.2%) developed mPM. Patients in the PM group presented higher preoperative carcinoembryonic antigen (CEA) [median (IQR): 4.5 (2.5–13.0) vs. 2.7 (1.5–5.9), *P* = 0.001] and CA 19-9 [median (IQR): 17.7 (12.0–37.0) vs. 10.8 (5.0–21.0), *P* = 0.001], advanced disease (pT4a 42.6% vs. 13.5%; pT4b 16.2% vs. 3.2%; *P* < 0.001), and negative pathological characteristics. Multivariate logistic regression identified CA 19-9, pT stage, pN stage, extent of lymphadenectomy, and lymphovascular invasion as significant predictors, and individual risk scores were calculated for each patient. The risk of recurrence increased remarkably with score values, and the model demonstrated a high negative predictive value (98.8%) and accuracy (83.9%) for scores below five.

**Conclusions:**

Besides confirming incidence and risk factors for mPM, our study developed a useful clinical tool for prediction of mPM risk. After external validation, this scoring system may guide personalized decision-making for patients with locally advanced CC.

**Supplementary Information:**

The online version contains supplementary material available at 10.1245/s10434-022-12097-9.

Despite the improvement in survival of patients with colon cancer (CC), 20–30% of patients still develop recurrent disease after curative resection.^1^ Peritoneal metastases (PM) are a rare event accounting for 3–6% of recurrences,^2^ but they are associated with dreadful prognosis.^3^ The standard therapy for metachronous PM (mPM) has not yet been established,^4^ and some authors are investigating the role of cytoreductive surgery (CRS) with or without hyperthermic intraperitoneal chemotherapy (HIPEC) to improve overall and disease-free survival in patients with localized PM.^5–8^ Similarly, randomized trials are underway to investigate the role of prophylactic second-look surgery and HIPEC.^9–11^ The efficacy of these strategies depends on the extent of peritoneal disease,^12^ therefore efforts should be made toward early identification of limited mPM or selection of patients at higher risk. Most of the previous reports on risk factors for PM are population-based^2,13–15^ or single-center^16^ studies, including patients who underwent noncurative resections,^17^ with synchronous PM, or systemic metastases.^18^ Several factors have been associated with increased risk of mPM, including the presence of locally advanced disease,^13,19,20^ but evidence derived from systematic reviews and meta-analysis confirmed a strong association only for synchronous PM, synchronous isolated ovarian metastases, and perforated primary tumor.^21,22^

The aim of this retrospective multicenter study was to assess the incidence of mPM in a large cohort of surgically resected CC, and to develop a scoring system on the basis of commonly used variables to identify patients at higher risk of peritoneal recurrence.

## Patients and Methods

### Study Design

This was an international multicenter retrospective cohort study that analyzed patients from five tertiary referral centers following the guidelines set out in the STROBE statement.^23^ Data were retrieved from each center’s database and shared with the promoting center in an anonymized spreadsheet. Guidelines of common definitions on tumor location, extent of surgery, and type of recurrence were formulated by the promoting center and circulated among the others. All methods used in this study were performed in accordance with the relevant ethical guidelines and regulations. The study protocol was approved by the institutional review board and ethic committee of each center if deemed necessary. All consecutive patients with confirmed pT3–4 CC operated between January 2014 and December 2018 were included. Both pT3 and pT4 were included since preoperative discrimination based on imaging modalities is currently unreliable and many cases are often under- or overstaged.^24,25^ Conversely, pT1–2 CC were excluded since previous literature showed that the risk of mPM in this subset is almost negligible.^13^

The primary outcome of the study was the rate of mPM after potentially curative surgery for CC. Secondary aims were the identification of significant risk factors and the development of a statistical model for the definition of mPM risk.

### Inclusion Criteria

Inclusion criteria were age equal to or greater than 18 years, histological diagnosis of pT3 or pT4 colonic adenocarcinoma, resection with curative intent (R0–R1 resection), and a minimum follow-up of 24 months. Rectal cancer, histotype other than adenocarcinoma, stage IV disease, patients who received neoadjuvant chemotherapy, palliative surgery (R2 resection), or concomitant tumors other than CC were excluded. Also, patients who died within 30 days from surgery were excluded from the computation of mPM.

### Data Collection

Each center retrieved data on demographic and clinical features, surgical procedure, postoperative course, and pathology. Preoperative value of carcinoembryonic antigen (CEA) and cancer antigen 19-9 (CA 19-9) were registered whenever available. Tumors were staged according to the 8th Edition of the AJCC Cancer Staging Manual.^26^ Morbidity was classified according to Clavien–Dindo. Clavien–Dindo grade I and II were grouped as mild complications, while grade III and IV as severe.^27^ Comorbidities were evaluated using the American Society of Anesthesiologists (ASA) Physical Status Classification and the Age-adjusted Charlson Comorbidity Index.^28^ Follow-up was conducted according to each center’s protocol and national guidelines. Retrieved data included status, cause of death, first site of recurrence, and treatment of recurrence. When more than one recurrence occurred at the same time, all sites of recurrence were registered. Peritoneal recurrence was diagnosed on the basis of radiological signs or clinical symptoms in patients candidates for systemic chemotherapy or best supportive care, while it was confirmed on pathological analysis for those who underwent exploratory surgery. Peritoneal recurrence was defined as isolated when the recurrence was limited to the peritoneum. Ovarian metastases were classified as PM.

### Statistical Analysis

Categorical data were compared using the chi-square test or Fisher’s exact test. Continuous data were analyzed using the analysis of variance (ANOVA) test or the Kruskal–Wallis test, as appropriate. Overall survival (OS) was defined as the length of time between primary surgery and time of death from any cause, while cancer-related survival (CRS) was considered death from cancer as the end point. Patients who died within 30 days from surgery were considered as postoperative deaths and were not included for the computation of recurrence and long-term survival. Time to peritoneal recurrence was calculated as time between primary surgery and the diagnosis of mPM, date of death, or end of follow-up, whichever occurred first. OS, CRS, and time to mPM were computed using the Kaplan–Meier method and compared by log-rank test. All tests were two-sided, and *P* < 0.05 was considered statistically significant.

For computation of the score, a logistic regression model was built as previously described in literature.^29^ Only patients with complete data on target covariates were included for development of the predicting score. Presence of mPM was the dependent variable, whereas clinical and pathologic variables were considered as numerical or categorical covariates. Numerical covariates were age (years) and surgical time (minutes). Categorical covariates were: CEA value (normal 0, elevated 1), CA 19-9 value (normal 0, elevated 1), surgical approach (open 0, laparoscopy 1), extent of lymphadenectomy (D1–2 0, D3 1), depth of tumor invasion (pT3 0, pT4a 1, pT4b 2), nodal status (pN0 0, pN1a 1, pN1b 2, pN1c 3, pN2a 4, pN2b 5), grading (G1–2 0, G3 1), lymphovascular invasion (absent 0, present 1), perineural invasion (absent 0, present 1), histological subtype (adenocarcinoma 0, mucinous carcinoma 1), and adjuvant chemotherapy (yes 0, no 1). The code “0” was assigned to the reference category. In the statistical program, the contrast for the comparison of categories was defined as “simple”; as such, each category of the predictor variable (except the reference category) was compared with the reference category. The parameters of the model were estimated using the maximum-likelihood method. Significant variables were included in the model by means of forward stepwise selection: starting with a model containing only the constant, the variable with the smallest significance value entered the model at each step, with a default level of *P* < 0.05. Significance value of each factor was reassessed at each step; if a variable in a forward stepwise block exceeded a significance level of 0.1, it was removed from the model. Removal testing was based on the probability of the likelihood-ratio statistic. The final model generated a set of independent prognostic variables, with their *β* regression coefficients, standard error (SE) of the coefficients, and *P* values. The fit of the model was verified by the Hosmer–Lemeshow goodness of fit test. The probability of the event (recurrence) was estimated by the formula: *e*^*z*^/(1 + *e*^*z*^) × 100, where *e* is the base of natural logarithm and *Z* is the result deriving from the logistic regression equation:

$$Z = c + B_{{1}} X_{{1}} + B_{{2}} X_{{2}} + \ldots B_{p} X_{p} ,$$

where *c* is the constant of the logistic regression model, and *X*_1_..._2_ are the independent variables identified by the model, with their regression coefficients (*B*_1_..._*p*_).^29^ With this method, we were able to estimate the probability of recurrence for each patient.

Statistical analysis was performed using SPSS software version 23.0 (IBM Corporation, Armonk, NY).

## Results

### Patient Selection

Of the 1454 patients who had surgery for CC at the participating centers, 28 were excluded owing to postoperative death (*n* = 3), discovery of unexpected synchronous metastases at the time of surgery (*n* = 14), noncurative resection (*n* = 2), or lack of adequate follow-up (*n* = 12). After the application of inclusion criteria, 1423 patients were analyzed. mPM was diagnosed in 74 patients (5.2%), and it represented the only site of recurrence in 45 patients (3.2%). Patients who developed mPM (PM group) were compared with patients who did not recur at the peritoneum (PM-free group).

### Survival and Recurrence

At the time of analysis, 110 patients (7.8%) had died. The remaining 1313 patients presented a median follow-up of 42 months (range 24–82 months). As expected, both 5-year OS (49.8% vs. 89.1%, *P* < 0.001) and CRS (56.9% vs. 96.5%, *P* < 0.001) were very poor in patients who developed mPM compared with PM-free patients.

**Fig. 1:**
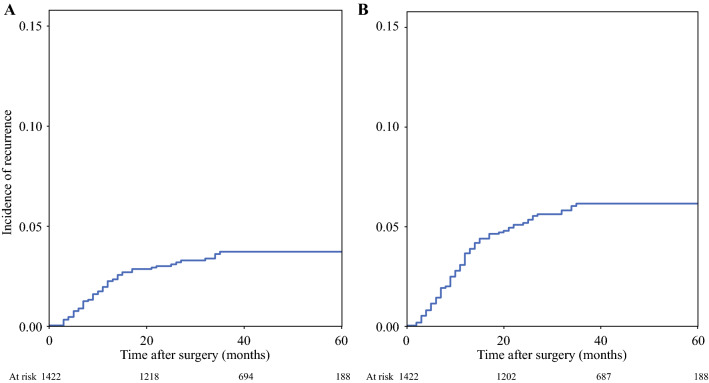
Kaplan–Meier estimates of the cumulative risk to develop metachronous peritoneal recurrence **A** isolated peritoneal recurrence (3.9%), **B** peritoneal recurrence (6.2%)

The 5-year cumulative risk to develop mPM was 6.2% (Fig. 1; isolated mPM 3.9%). mPM developed after a mean ± SD time of 12.6 ± 4.3 months (median: 11 months, range 3–35 months), and mean ± SD survival after diagnosis of mPM was 16.8 ± 14.4 months (median: 14 months, range 0–67 months). Data on treatment of isolated mPM was available for 38 of 45 patients: 18 patients received systemic chemotherapy, 7 underwent surgery, 7 received CRS with concomitant HIPEC, and 6 were addressed to best supportive care.

### Cohort Characteristics

Patients who developed mPM showed similar characteristics in terms of gender, BMI, and comorbidity status, while they were older than patients in the PM-free group (Table 1). Interestingly, the PM group showed higher preoperative levels of both CEA [median (IQR): 4.5 (2.5–13.0) vs. 2.7 (1.5–5.9), *P* = 0.001] and CA 19-9 [median (IQR): 17.7 (12.0–37.0) vs. 10.7 (5.0–21.0), *P* = 0.001]. Patients who developed mPM presented more often with obstruction (4.1% vs. 2.3%) and perforation (2.7% vs. 1.7%), but the difference was not statistically significant.

**Table 1:** Patients demographics and clinical characteristics according to recurrence status

	Total (*n* = 1423)	PM-free (*n* = 1349)	PM (*n* = 74)	*P*-value
Age, mean ± SD (years)	1423	66.5 ± 12.5	67.9 ± 10.9	**0.028**
Gender, male	1423	711 (52.7)	42 (56.8)	0.551
ASA physical status class ≥ 3	1421	265 (19.7)	22 (29.7)	0.052
*BMI (kg/m*^*2*^*)*	1413			0.804
Normal (< 25)		886 (66.1)	51 (69.9)	
Overweight (25–29.9)		374 (27.9)	18 (24.7)	
Obese (≥ 30)		80 (6.0)	4 (5.5)	
CACI, mean ± SD	1221	4.2 ± 1.7	4.4 ± 1.4	0.339
Median preoperative CEA (IQR), ng/mL	1341	2.7 (1.5–5.9)	4.5 (2.5–13.0)	**0.001**
Preoperative CEA, elevated	384/1341	353 (27.7)	31 (45.6)	**0.002**
Median preoperative CA 19-9 (IQR), U/mL	1194	10.7 (5.0–21.0)	17.7 (12.0–37.0)	**0.001**
Preoperative CA 19-9, elevated	243/1194	222 (19.4)	21 (40.4)	**0.001**
Preoperative obstruction	34/1423	31 (2.3)	3 (4.1)	0.417
Preoperative perforation	25/1422	23 (1.7)	2 (2.7)	0.376

Number in parentheses are percentages unless otherwise specified

*P* < 0.05 are highlighted in bold

*SD* standard deviation, *IQR* interquartile range, *BMI* body mass index, *CACI* age-adjusted Charlson Comorbidity Index, *CEA* carcinoembryonic antigen, *CA 19-9* cancer antigen 19-9

With regard to pathological characteristics (Table 2), the PM group showed a higher percentage of advanced stages (stage IIIC 35.1% vs. 8.5%, *P* < 0.001), and a greater rate of pT4 tumors (pT4a 42.6% vs. 13.5%; pT4b 16.2% vs. 3.2%; *P* < 0.001) and lymph node metastases (pN1 48.6% vs. 30.9%; pN2 32.4% vs. 14.7%; *P* < 0.001). Although the rate of R0 resections was similar, infiltration of radial margin occurred more often in the PM group (4.1% vs. 0.8%, *P* = 0.007). Finally, the PM group presented more frequently with poorly differentiated grading (28.8% vs. 14.3%, *P* = 0.004), lymphovascular invasion (74.0% vs. 44.8%, *P* < 0.001), and perineural invasion (68.5% vs. 43.0%, *P* < 0.001).

**Table 2:** Pathological characteristics according to development of peritoneal recurrence

	Total (*n* = 1423)	PM-free (*n* = 1349)	PM (*n* = 74)	*P*-value
*Tumor location*				0.339
Right colon	634	597 (44.3)	37 (50.0)	
Left colon	789	752 (55.7)	37 (50.0)	
*TNM stage*				**< 0.001**
IIA	654	649 (48.1)	5 (6.8)	
IIB	69	64 (4.7)	5 (6.8)	
IIC	24	20 (1.5)	4 (5.4)	
IIIB	536	502 (37.2)	34 (45.9)	
IIIC	140	114 (8.5)	26 (35.1)	
*Infiltration of the bowel wall*				**< 0.001**
pT3	1153	1124 (83.3)	29 (39.2)	
pT4a	215	182 (13.5)	33 (42.6)	
pT4b	55	43 (3.2)	12 (16.2)	
*Lymph-nodes status*				**< 0.001**
pN0	748	734 (54.4)	14 (18.9)	
pN1	453	417 (30.9)	36 (48.6)	
pN2	222	198 (14.7)	24 (32.4)	
R0 resection	1414	1326 (99.0)	71 (95.9)	0.055
Infiltration of radial margin	14/1240	11 (0.8)	3 (4.1)	**0.008**
Harvested LN, median (IQR)	1423	28 (21–37)	28 (19–37)	0.456
Positive LN, median (IQR)	1423	0 (0–2)	2 (1–4)	**< 0.001**
Less than 12 LN	1423	37 (2.7)	3 (4.1)	0.459
Tumor size, mean ± SD, cm	1423	50.1 ± 34.8	54.9 ± 18.5	0.079
*Histologic grade*	1385			**0.003**
Well-differentiated	65	62 (4.7)	3 (4.1)	
Moderate	1111	1062 (81.0)	49 (67.1)	
Poorly differentiated	209	188 (14.3)	21 (28.8)	
Mucinous histology	104/1423	95 (7.0)	9 (12.2)	0.278
Lymphovascular invasion (LVI)	656/1418	602 (44.8)	54 (74.0)	**< 0.001**
Perineural invasion (PNI)	619/1395	569 (43.0)	50 (68.5)	**< 0.001**
Presence of budding	409/974	385 (41.9)	24 (43.6)	0.888

Number in parentheses are percentages unless otherwise specified

*P* < 0.05 are highlighted in bold

*SD* standard deviation, *IQR* interquartile range, *LN* lymph nodes

Laparoscopy was the preferred approach in both groups (Table 3). A lower proportion of patients in the PM group underwent laparoscopic surgery compared with the PM-free group (68.9% vs. 84.1%, *P* = 0.003), and the conversion rate was higher (2.7% vs. 1.7%). With regard to operative outcomes, patients in the PM group underwent longer surgeries (mean operative time: 186.4 ± 93.9 min vs. 152.4 ± 75.9 min, *P* = 0.002) despite a lower rate of extended lymphadenectomies (63.0% vs. 77.7%, *P* = 0.013). Complication rates did not differ between groups, while postoperative length of stay was longer in the PM group [median postoperative length of stay: 8 (6–10) vs. 7 (6–9), *P* = 0.021]. Adjuvant chemotherapy was administered in more than half of the patients in both groups, and more frequently in those who developed mPM (70.3% vs. 58.7%, *P* = 0.052). Standard chemotherapy regimens included: XELOX (*n* = 257, 30.9%), FOLFOX/FOLFIRI (*n* = 326, 39.1%), capecitabine alone (*n* = 224, 26.9%), 5-fluorouracil alone (*n* = 5, 0.6%), and other schemes (*n* = 22, 2.6%).

**Table 3:** Comparison of surgical data and short-term outcomes

	Total (*n* = 1423)	PM-free (*n* = 1349)	PM (*n* = 74)	*P*-value
*Operative method*				**0.003**
Open	213	192 (14.2)	21 (28.4)	
Laparoscopic	1185	1134 (84.1)	51 (68.9)	
Converted	25	23 (1.7)	2 (2.7)	
Operative time, mean ± SD	1423	152.4 ± 75.9	186.4 ± 93.9	**0.002**
Extended lymphadenectomy (D3)	1083/1408	1037 (77.7)	46 (63.0)	**0.013**
Severe postoperative complications	56/1423	50 (3.7)	4 (5.4)	0.124
*Postoperative complications*	212/1423			
Anastomotic leak	27	24 (1.8)	3 (4.1)	0.174
SSI	49	46 (3.5)	3 (4.0)	0.357
PPOI	56	55 (4.1)	1 (1.4)	0.360
Median postoperative length of stay (IQR)	1423	7 (6–9)	8 (6–10)	**0.021**
Adjuvant chemotherapy	838/1413	786 (58.7)	52 (70.3)	0.052

Number in parentheses are percentages unless otherwise specified

*P* < 0.05 are highlighted in bold

*SD* standard deviation; *IQR* interquartile range

### Risk Factors for mPM

The final model identified elevated CA 19-9, nodal status, depth of tumor invasion, lymphovascular invasion, and extent of lymphadenectomy as independent predictors of recurrence; the corresponding *β* regression coefficients, SE of the coefficients, and *P*-values are reported in Table 4 and visually depicted in Supplementary Fig. 1. The Hosmer–Lemeshow goodness of fit test of the final model was *P* = 0.520, thus indicating that the model fit data adequately.

**Table 4:** Independent predictors of peritoneal recurrence (logistic regression analysis)

Variable	*P*-value	HR	95% CI	Coefficient	SE of the coefficient
Elevated CA 19-9	0.003	2.8	1.4–5.4	1.016	0.339
Extended lymphadenectomy (D3)	0.046	0.5	0.2–0.9	−0.732	0.367
Lymphovascular invasion	0.047	2.0	1.1–3.8	0.677	0.341
*Depth of tumor invasion*	< 0.001				
pT3		–	–		
pT4a		8.3	4.2–16.5	2.117	0.350
pT4b		12.9	4.7–35.7	2.557	0.519
*Lymph nodes metastases*	0.044				
pN0		–	–		
pN1a		2.6	1.0–6.9	0.975	0.492
pN1b		2.7	1.0–7.0	0.995	0.488
pN1c		7.7	1.7–35.1	2.043	0.774
pN2a		1.8	0.6–5.5	0.603	0.560
pN2b		4.2	1.4–12.1	1.432	0.543

**Fig. 2:**
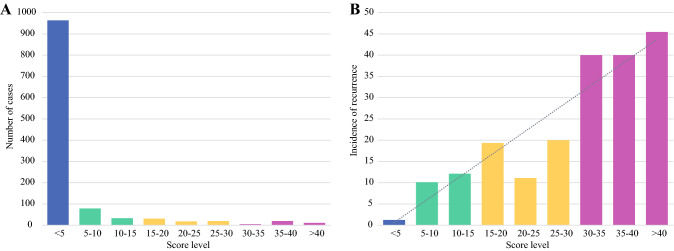
**A** Distribution of score values in patients included in the analysis, **B** incidence of recurrence in score subgroups

**Fig. 3:**
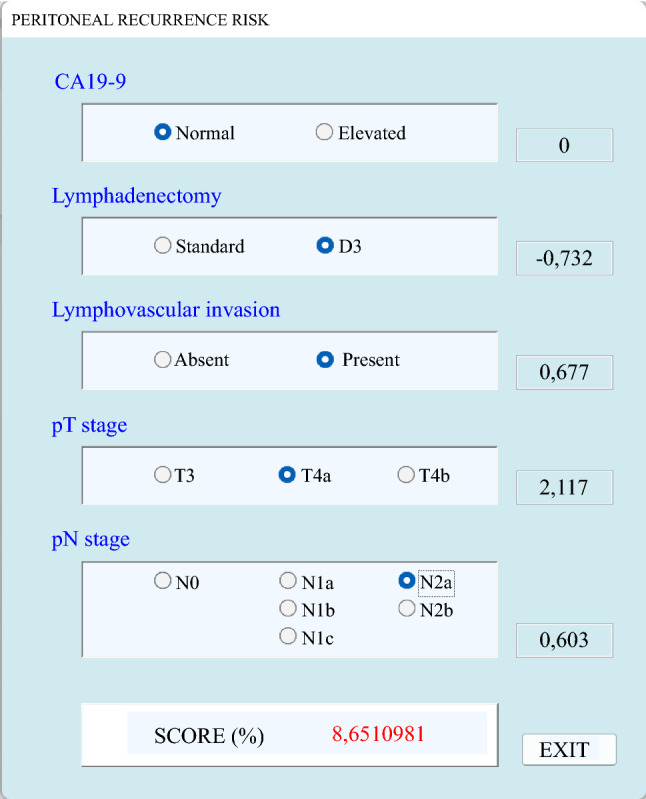
Custom-made score calculator based on factors identified at logistic regression

Score values were obtained for all patients with complete data on relevant variables. The distribution of the score is reported in Fig. 2A, while the incidence of mPM according to score subgroups is shown in Fig. 2B. Values ranged between 0.32 and 55.6 and were stratified into nine subgroups. Most patients (920 cases, 76.8%) had a value below five. Notably, the percentage of patients developing mPM in this group was 1.2%, hence the negative predictive value with a score below 5 was 98.8% (95% CI, 98.0–99.2%). Overall accuracy for prediction of mPM with a score above 5 was 83.9%. The risk of recurrence increased proportionally with score values, reaching 40% in patients with scores 30–40, and 45% in patients with scores above 40 (Fig. 2B; Supplementary Fig. 2). Area under receiver operating characteristic (AUC ROC) (Supplementary Fig. 3) for the risk score was 0.867 (SE 0.025, 95% CI, 0.818–0.917). The values of AUC did not differ significantly when stratifying the analysis according to clinical variables not included in the model (Supplementary Table 1). Interestingly, the highest value of AUC was found in the subgroup of patients with mucinous histology (AUC 0.934). In this latter group, none of the patients with a risk score below 20 developed mPM. A clinical calculator for the prediction of mPM risk was developed (Fig. 3) for external validation, and it can be downloaded from Supplementary Materials (PM score calculator).

## Discussion

Epidemiological data on the real incidence of mPM are scarce, with data reported in literature ranging from 2 to 20%.^13,18,30,31^ Most data derive from retrospective population-based databases which included patients with stage IV disease, synchronous PM, and patients treated before the advent of modern chemotherapy regimens.^2,3,13,18^ Part of the uncertainty about prevalence of mPM derives from the challenges of early-stage diagnosis and quantification, given that computed tomography (CT) as well as magnetic resonance imaging (MRI) present limited sensitivity.^32,33^ The results of our large multicenter international analysis confirmed that the risk to develop mPM at 5 years after curative surgery for CC settles at 6.2%, which is in line with most recent literature.^2,13,15,16,34,35^

A systematic review on risk factors for mPM conducted by Honoré et al. identified three clinical scenarios presenting higher risk: synchronous resected PC, presence of ovarian metastases, and perforated primary tumors.^21^ Evidence for other risk factors was poor, but the authors recognized as potential prognosticators histological subtype, serosal invasion, positive peritoneal lavage, and lymph node metastases.^21^ More recently, Zhang et al. conducted a systemic review and meta-analysis and found that mPM was positively associated with tumor perforation, poor differentiation, pT4, lymph node metastases, mucinous histology, obstruction, synchronous ovarian metastases, elevated tumor markers, and positive peritoneal citology.^22^ Although 36 articles were included, the meta-analysis may be underpowered as it consisted of a limited number of studies, ranging from 2 to 12 for each risk factor. Nonetheless, our study showed a relevant association between mPM and elevated preoperative CEA and CA 19-9, preoperative obstruction, advanced tumor stage represented by both serosal invasion (pT4) and lymph node metastases, infiltration of circumferential resection margins, poor differentiation, lymphovascular invasion, and perineural invasion. Tumor perforation was more frequent in the PM group (4.9% vs. 2.9%), but the difference was not statistically significant, most likely owing to the small number of events in our cohort. With regard to peritoneal lavage, it is not routine practice in our centers, therefore no conclusions can be drawn on this aspect.

Our secondary aim was to build a statistical model for the definition of risk of recurrence at the peritoneum. Although many risk factors have been stochastically considered, the actual weight of each variable on the probability of recurrence remains obscure. Multivariate logistic regression analysis was conducted to identify statistically significant risk factors. In our model (Fig. 3), the risk of mPM was significantly associated with elevated preoperative CA 19-9, limited lymphadenectomy, depth of tumor infiltration, lymph node metastases, and lymphovascular invasion (Supplementary Fig. 1). The strongest risk factors were pT4a and pT4b stages, and the presence of free tumor deposits (pN1c). Interestingly, 76.8% of patients presented a score below 5 (Fig. 2A), which had an exceptionally high negative predictive value (98.8%) for the development of mPM. This score is relevant in that it could exclude development of mPM in more than three-quarters of patients with only 1% risk of error. Our results may also put into question the calculation of the sample size of ongoing and recently published randomized studies that investigated the role of prophylactic second-look surgery and HIPEC in high-risk patients.^5,9,10^ Although the PHOPYLOCHIP-PRODIGE 15 trial only enrolled patients with synchronous resected minimal PM, resected ovarian metastases, and tumor perforation,^9^ the COLOPEC^10^ and the HIPECT4^5^ studies comprise patients with clinical T4, with inclusion criteria similar to our analysis. Involvement of all patients with clinical locally advanced tumors may explain the lack of efficacy of treatments in these studies, since three-quarters of patients presented a negligible risk to develop mPM.

A high correspondence between the score and prognosis was observed. Indeed, patients with a score above 30 showed a risk of recurrence of 40–45% (Fig. 2B; Supplementary Fig. 2), which is well above the median value reported in literature for CC.^2,13,16,34,35^ Such a predictive scoring system based on commonly available clinicopathological variables could be used for tailored intensive follow-up strategies or application of proactive strategies in selected high-risk patients. It is well known that the extent of peritoneal disease^12^ and R0 resection have the highest prognostic significance in the treatment of PM.^12,36,37^ Early diagnosis of mPM through noninvasive methods remains a diagnostic challenge, as many patients may be asymptomatic or without radiological signs of recurrence.^32,33,38^ To overcome this limitation, some proactive strategies have been developed for early detection or prevention of mPM, such as planned second-look surgery plus HIPEC. However, these invasive approach may be burdened by significant complications^9,10^ and should only be offered to a restricted cohort of high-risk patients,^22,37^ potentially identified through predictive risk scores such as the one we developed. Although the predictive score shows good concordance with the risk of recurrence, other covert factors should be investigated to fully explain the predisposition of some patients to recur at the peritoneum. The identification of high-risk patients may prompt further targeted studies evaluating the role of histological subtypes and molecular biology in the development of PM.

Risk prediction systems, nomograms, and statistical models have increasingly been used in clinical oncology to predict the risk of recurrence or long-term outcomes.^29,39–42^ However, only one Swedish group has conducted two studies to develop a tool for predicting mPM in patients with CC. First, they derived two prediction models for colon and rectal cancer using data extracted from a cancer registry. Factors included were age, pT stage, pN stage, number of examined lymph nodes, type of surgery, completeness of cancer resection, adjuvant chemotherapy, and tumor location. The resulting risk scores were grouped in quartiles and showed good agreement with observed probability of mPM.^17^ They conducted a second analysis to externally validate the predictive risk model on a validation cohort obtained from the population-based Swedish Colorectal Cancer Registry. When applied to the new validation dataset, the model predicted mPM with a concordance index of 79%.^43^ These two studies presented a large sample size and good internal validity. However, some limitations should be acknowledged, including the remote period of accrual (1995–2007 for the original cohort, 2008–2010 for the validation cohort) and the use of registry-based data. Moreover, the authors comprised patients who underwent noncurative (R2) resection, which is a known risk factor for poor prognosis and disease progression.^44,45^

Our study presents some limitations that should be recognized. Because of its retrospective nature, it was not possible to retrieve complete data on some variables, specifically preoperative tumor markers and molecular characterization. Although not routinely assessed, nonmetastatic CC, *RAS* and *BRAF*^46^ mutations as well as other gene-based molecular profiles^47^ may account for part of the unexplained remaining risk of PM. Since the determination of such features could be technically complex and frequently presents high costs, the identification of patients at higher risk may enable targeted molecular, genetic, and immunohistochemistry studies with resulting contained costs. Since mPM represent a rare event for patients with CC, we decided to use the whole cohort for the development of the prediction model, without splitting patients into a training and validation set. Hence, the efficacy of the proposed risk prediction model should be validated in an external cohort. Finally, the predictive score is mainly composed of postoperative variables (pT, pN, extent of lymphadenectomy, presence of lymphovascular invasion), limiting its application in preoperative selection of patients who may benefit from synchronous multimodal treatments.

Notwithstanding these limitations, our scoring system is one of the few available tools for the prediction of individual risk of mPM after potentially curative surgery for locally advanced CC. It is built on easily available clinical and pathological variables, and it represents a practical instrument for identification of high-risk patients. More importantly, it excludes with 98.8% certainty the development of mPM in more than 75% of the patients. The multicentric nature of the study, the surgical expertise and volume of the participating centers, and the strict inclusion criteria add to the strengths and generalizability of our analysis.

## Conclusions

Relapse as PM is a rare event in patients after curative resection for CC, and its occurrence is associated with elevated preoperative tumor markers, advanced tumor stage, infiltration of the radial margin, and negative pathological prognostic factors. Individual risk of recurrence calculated using our prediction model showed good concordance with risk of recurrence and a high negative predictive value for scores below five, representing a useful tool for personalized decision-making.

## Supplementary Information

Below is the link to the electronic supplementary material.

Supplementary file1 (PDF 412 KB)

Supplementary file2 (PDF 397 KB)

Supplementary file3 (PDF 117 KB)

Supplementary file4 (PDF 83 KB)

Supplementary file5 (DOCX 15 KB)

Supplementary file6 (DOCX 33 KB)
