# Supplementary material for: Prediction of Metachronous Peritoneal Metastases After Radical Surgery for Colon Cancer: A Scoring System Obtained from an International Multicenter Cohort
Source: Ann Surg Oncol. 2022 Jul 5;29(12):7896–906. doi: 10.1245/s10434-022-12097-9 (PMC9550705; doi:10.1245/s10434-022-12097-9)
Supplement: Supplementary file 2 — Supplementary file2 (PDF 397 KB) [file 10434_2022_12097_MOESM2_ESM.pdf]

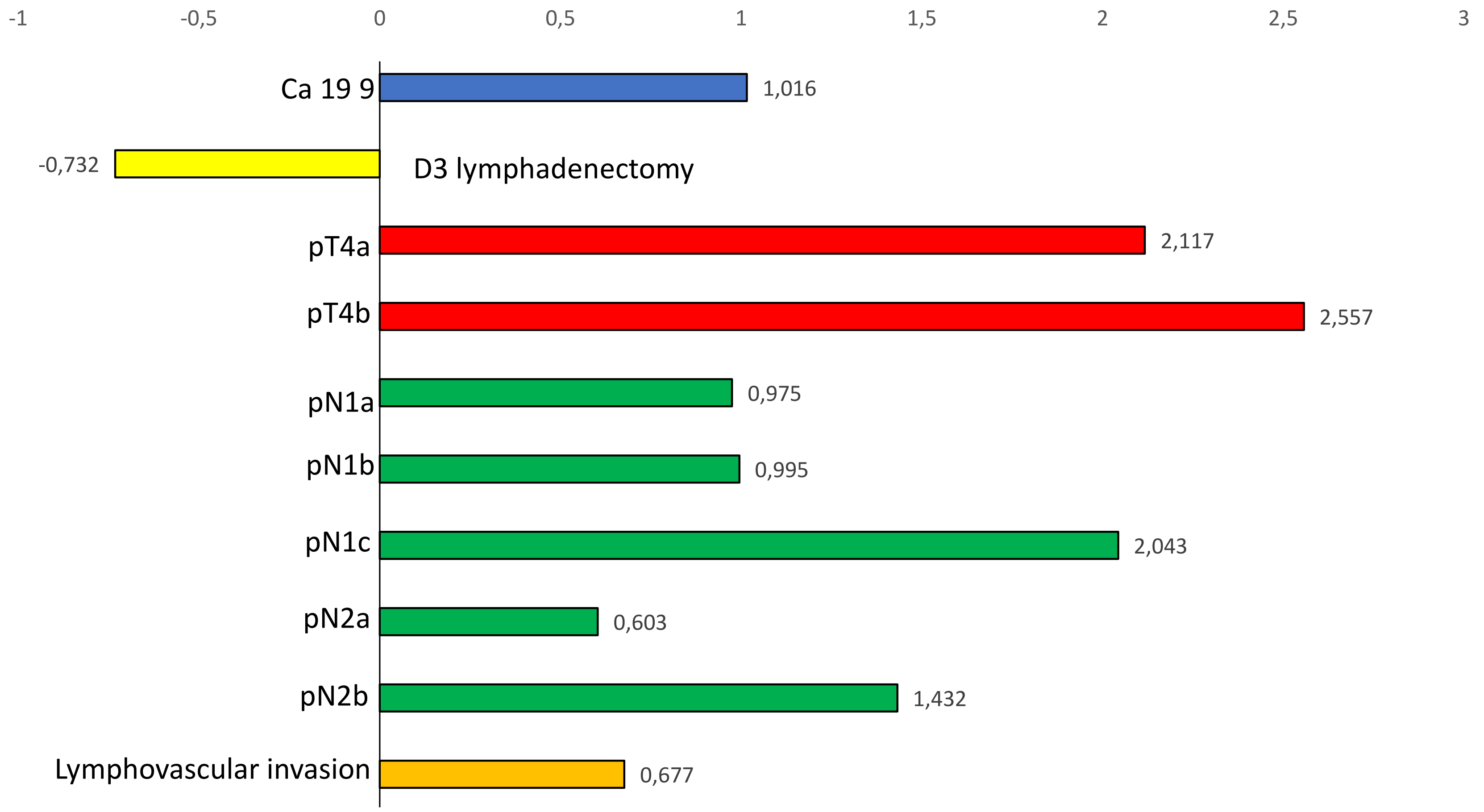

Supplementary Figure 1. Visual representation of the weight of the single variables on the computation of the risk score.
