# Supplementary material for: Prediction of Metachronous Peritoneal Metastases After Radical Surgery for Colon Cancer: A Scoring System Obtained from an International Multicenter Cohort
Source: Ann Surg Oncol. 2022 Jul 5;29(12):7896–906. doi: 10.1245/s10434-022-12097-9 (PMC9550705; doi:10.1245/s10434-022-12097-9)
Supplement: Supplementary file 3 — Supplementary file3 (PDF 117 KB) [file 10434_2022_12097_MOESM3_ESM.pdf]

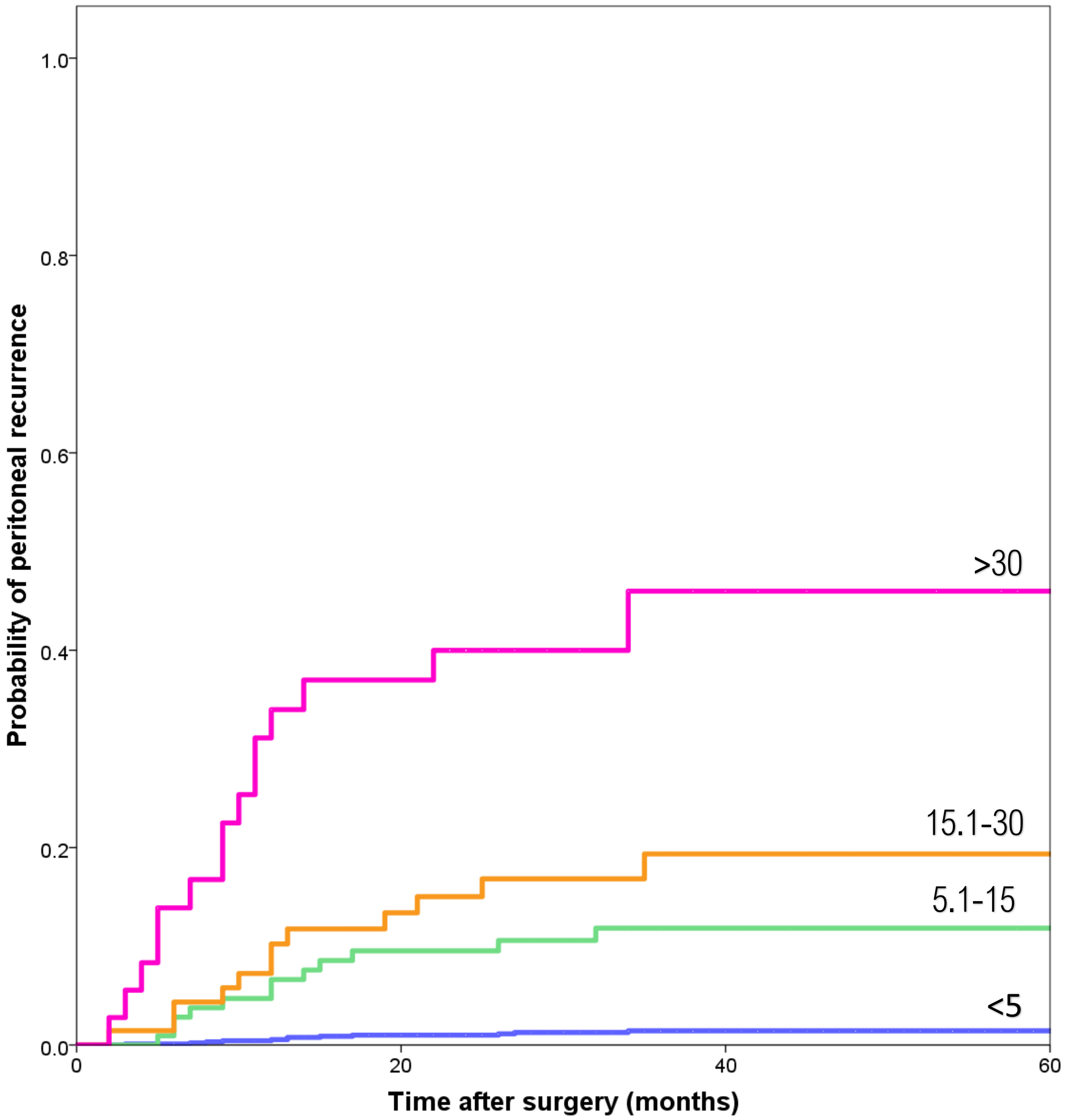

Supplementary Figure 2. Kaplan-Meier estimates of the incidence of peritoneal recurrence according to score groups.
