# Supplementary material for: Prediction of Metachronous Peritoneal Metastases After Radical Surgery for Colon Cancer: A Scoring System Obtained from an International Multicenter Cohort
Source: Ann Surg Oncol. 2022 Jul 5;29(12):7896–906. doi: 10.1245/s10434-022-12097-9 (PMC9550705; doi:10.1245/s10434-022-12097-9)
Supplement: Supplementary file 5 — Supplementary file5 (DOCX 15 KB) [file 10434_2022_12097_MOESM5_ESM.docx]

| **Variable** | | **Area (95% CI)** | **Standard error** | **Asymptotic significance** |
| --- | --- | --- | --- | --- |
| Age | |  |  |  |
|  | <70 | 0.877 (0.809 – 0.945) | 0.035 | <0.001 |
|  | ≥70 | 0.858 (0.786 – 0.930) | 0.037 | <0.001 |
| Gender | |  |  |  |
|  | Male | 0.888 (0.833 – 0.943) | 0.028 | <0.001 |
|  | Female | 0.839 (0.750 – 0.928) | 0.045 | <0.001 |
| Tumor location | |  |  |  |
|  | Right colon | 0.848 (0.758 – 0.938) | 0.046 | <0.001 |
|  | Left colon | 0.883 (0.831 – 0.935) | 0.026 | <0.001 |
| Type of surgery | |  |  |  |
|  | Open | 0.877 (0.761 – 0.993) | 0.059 | 0.001 |
|  | Laparoscopic | 0.865 (0.810 – 0.920) | 0.028 | <0.001 |
| Histology | |  |  |  |
|  | Mucinous | 0.934 (0.870 – 0.999) | 0.033 | 0.038 |
|  | Non mucinous | 0.865 (0.814 – 0.916) | 0.026 | <0.001 |
| Perineural invasion | |  |  |  |
|  | Present | 0.842 (0.784 – 0.901) | 0.030 | <0.001 |
|  | Absent | 0.851 (0.741 – 0.962) | 0.056 | <0.001 |
| Grading | |  |  |  |
|  | G1-2 | 0.857 (0.794 – 0.921) | 0.032 | <0.001 |
|  | G3 | 0.850 (0.771 – 0.929) | 0.040 | <0.001 |
| Post-operative complications | |  |  |  |
|  | Yes | 0.839 (0.724 – 0.954) | 0.058 | 0.001 |
|  | No | 0.871 (0.815 – 0.928) | 0.029 | <0.001 |
| Adjuvant chemotherapy | |  |  |  |
|  | Yes | 0.870 (0.817 – 0.922) | 0.027 | <0.001 |
|  | No | 0.852 (0.743 – 0.962) | 0.056 | <0.001 |

**Supplementary Table 1.** ROC curve analysis and corresponding AUC for variables not included in the predictive scoring system.
